# Bis[μ-2-(4-hy­droxy­phen­yl)acetato]-κ^3^ *O*,*O*′:*O*;κ^3^ *O*:*O*,*O*′-bis­{aqua­(4,4′-bipyridine-κ*N*)[2-(4-hy­droxy­phen­yl)acetato-κ^2^ *O*,*O*′]samarium(III)} monohydrate

**DOI:** 10.1107/S1600536810044454

**Published:** 2010-11-06

**Authors:** Jia-Lu Liu, Jian-Feng Liu, Guo-Liang Zhao

**Affiliations:** a College of Chemistry and Life Sciences, Zhejiang Normal University, Jinhua 321004, People’s Republic of China, and Zhejiang Normal University Xingzhi College, Jinhua 321004, People’s Republic of China

## Abstract

The dinuclear title complex, [Sm_2_(C_8_H_7_O_3_)_6_(C_10_H_8_N_2_)_2_(H_2_O)_2_]·H_2_O, contains two Sm^III^ atoms, six deprotonated *p*-hy­droxy­phenyl­acetic acid (PAA) mol­ecules, two 4,4′-bipyridine (bipy) mol­ecules, two coordinated water mol­ecules and one solvent water molecule. Each Sm^III^ion is nine-coordinated by seven O atoms from four PAA ligands, one water O atom and one N atom from a bipy ligand in a distorted geometry. The PAA ligands are coordinated to the Sm^III^ ion in bridging and bridging tridentate modes. The asymmetric unit also contains one uncoordinated water mol­ecule. The occurrence of numerous O—H⋯O and O—H⋯N hydrogen bonds involving coordinated and non-coordinated water mol­ecules builds up an intricate three-dimensional network.

## Related literature

For related structures, see: Arias *et al.* (2000 ▶); Liu *et al.* (2010 ▶). For applications of carb­oxy­lic metal-organic complexes, see: Wang & Sevov (2008 ▶); Wang *et al.* (2010 ▶); Fang & Zhang (2006 ▶).
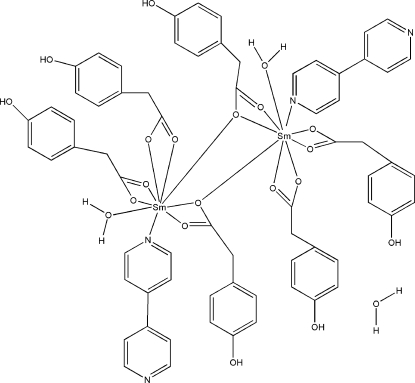


## Experimental

### 

#### Crystal data

[Sm_2_(C_8_H_7_O_3_)_6_(C_10_H_8_N_2_)_2_(H_2_O)_2_]·H_2_O
*M* *_r_* = 1573.96
Triclinic, 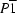

*a* = 11.7589 (3) Å
*b* = 16.3141 (4) Å
*c* = 18.4618 (5) Å
α = 83.619 (1)°
β = 72.025 (1)°
γ = 70.941 (1)°
*V* = 3183.90 (14) Å^3^
*Z* = 2
Mo *K*α radiation
μ = 1.91 mm^−1^
*T* = 296 K
0.34 × 0.14 × 0.03 mm

#### Data collection

Bruker APEXII area-detector diffractometer
Absorption correction: multi-scan (*SADABS*; Sheldrick, 1996 ▶) *T*_min_ = 0.733, *T*_max_ = 0.941
49293 measured reflections
14715 independent reflections
11851 reflections with *I* > 2σ(*I*)
*R*_int_ = 0.047

#### Refinement

*R*[*F* ^2^ > 2σ(*F* ^2^)] = 0.029
*wR*(*F* ^2^) = 0.070
*S* = 1.02
14715 reflections
875 parameters
9 restraints
H atoms treated by a mixture of independent and constrained refinement
Δρ_max_ = 0.66 e Å^−3^
Δρ_min_ = −0.72 e Å^−3^

### 

Data collection: *APEX2* (Bruker, 2006 ▶); cell refinement: *SAINT* (Bruker, 2006 ▶); data reduction: *SAINT*; program(s) used to solve structure: *SHELXS97* (Sheldrick, 2008 ▶); program(s) used to refine structure: *SHELXL97* (Sheldrick, 2008 ▶); molecular graphics: *SHELXTL* (Sheldrick, 2008 ▶); software used to prepare material for publication: *SHELXL97*.

## Supplementary Material

Crystal structure: contains datablocks I, global. DOI: 10.1107/S1600536810044454/kj2162sup1.cif

Structure factors: contains datablocks I. DOI: 10.1107/S1600536810044454/kj2162Isup2.hkl

Additional supplementary materials:  crystallographic information; 3D view; checkCIF report

## Figures and Tables

**Table 1:** Hydrogen-bond geometry (Å, °)

*D*—H⋯*A*	*D*—H	H⋯*A*	*D*⋯*A*	*D*—H⋯*A*
O3—H3*B*⋯O12^i^	0.82	1.94	2.749 (3)	169
O6—H6*B*⋯O3*W*^ii^	0.82	1.86	2.642 (3)	159
O9—H9*A*⋯O17^iii^	0.82	1.86	2.673 (3)	173
O12—H12*A*⋯O11^iv^	0.82	1.94	2.746 (3)	168
O15—H15*C*⋯O6^v^	0.82	1.91	2.722 (3)	174
O18—H18*B*⋯O9^ii^	0.82	1.95	2.768 (3)	173
O2*W*—H2*WA*⋯O5	0.83 (5)	2.02 (5)	2.767 (3)	150 (4)
O2*W*—H2*WB*⋯N2^ii^	0.83 (2)	2.03 (2)	2.841 (3)	164 (5)
O3*W*—H3*WB*⋯O3	0.85 (4)	1.99 (2)	2.816 (3)	167 (5)
O1*W*—H1*WA*⋯O13	0.81 (5)	1.96 (4)	2.755 (3)	165 (4)
O1*W*—H1*WB*⋯N4^i^	0.83 (2)	1.99 (2)	2.778 (3)	160 (4)
O3*W*—H3*WA*⋯O1^vi^	0.84 (4)	1.94 (4)	2.773 (3)	172 (4)

Symmetry codes: (i) 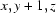; (ii) 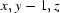; (iii) 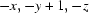; (iv) 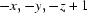; (v) 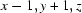; (vi) 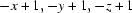.

**Fig. 1.**
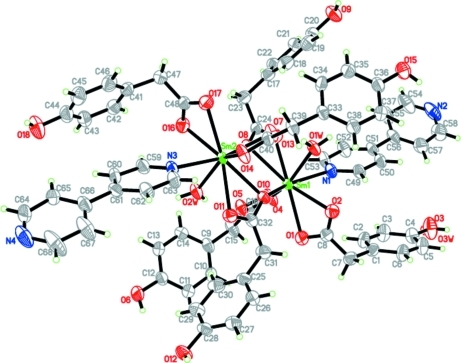
The molecular structure of the title complex, showing the atom-labelling scheme. Displacement ellipsoids are drawn at the 50% probability level.

## supplementary crystallographic information

### Comment

The design and synthesis of carboxylic metal-organic complexes have been an increasing interest for decades owing to their potential practical applications, such as fluorescence and magnetism (Wang, *et al.*, 2010; Fang *et al.*, 2006; Wang, *et al.*, 2008). We have published on this subject before (Liu, *et al.*, 2010). In the current paper we report the crystal structure of a new Sm^III^ complex with the ligand *p*- hydroxyphenylacetic acid. The title compound consist of six *L* ligands, two bipy molecules and two coordinated water molecules. In the dinuclear compound, each Sm ion is coordinated with seven O atoms from four *L* ligands, one N atom from 4,4'-bipyridine ligand and one O atom from a water molecule. The central Sm atom is nine coordinated. The PAA ligands are coordinating in two modes, bridging and bridging tridentate. (Fig.1). The Sm^III^ ion is in a distorted ball coordination geometry. The asymmetric unit further contains one solvent water molecule. Bond lengths and bond angles involving the metals and O atoms skeleton compare well with related structure as bis((µ2-Acetato-*O*,*O*,*O*')-diaqua-bis(acetato-O,*O*') -samarium(iii))pentahydrate (Arias, *et al.*, 2000), In addition, numerous O—H···O bonds involving both hydroxyl groups as well as coordinated and non-coordinated water molecules build up an intricate threedimensional network (Table 1).

### Experimental

All reagents and solvents used were of commercially available quality and were used without prior purification. *p*-hydroxyphenylacetic acid(HPAA) (0.456 g, 3 mmol) and sodium hydroxide (0.12 g, 3 mmol) were mixed together in water (10 ml). Sm(NO_3_)_3_ (0.336 g, 1 mmol), dissolved in water (10 ml), was added into the first solution. After stirring for an hour, an ethanol (5 ml) solution of 4,4'-bipyridine (0.156 g, 1 mmol) was slowly dripped into the mixture, followed by three hours stirring. After filtration, the filtrate was allowed to stand at room temperature and single crystals suitable for *X*–ray diffraction were obtained after a week.

### Refinement

All H atoms attached to C atoms and *O*(hydroxyl) atom were fixed geometrically and treated as riding with C—H = 0.97 Å (methylene) or 0.93 Å (aromatic) and O—H = 0.82 Å with *U*_iso_(H) = 1.2*U*_eq_(C) or *U*_iso_(H) = 1.5*U*_eq_(O). H atoms of water molecule were located in a difference Fourier map and included in the subsequent refinement using restraints (O—H= 0.82 (1)Å and H···H= 1.39 (2) Å) with *U*_iso_(H) = 1.5*U*_eq_(O). In the last cycles of refinement they were treated as riding on their parent O atom.

### Crystal data

**Table:**

[Sm_2_(C_8_H_7_O_3_)_6_(C_10_H_8_N_2_)_2_(H_2_O)_2_]·H_2_O	*Z* = 2
*M**_r_* = 1573.96	*F*(000) = 1584
Triclinic, *P*1	*D*_x_ = 1.642 Mg m^−^^3^
Hall symbol: -P 1	Mo *K*α radiation, λ = 0.71073 Å
*a* = 11.7589 (3) Å	Cell parameters from 9956 reflections
*b* = 16.3141 (4) Å	θ = 1.7–27.7°
*c* = 18.4618 (5) Å	µ = 1.91 mm^−^^1^
α = 83.619 (1)°	*T* = 296 K
β = 72.025 (1)°	Block, colourless
γ = 70.941 (1)°	0.34 × 0.14 × 0.03 mm
*V* = 3183.90 (14) Å^3^	

### Data collection

**Table:**

Bruker APEXII area-detector diffractometer	14715 independent reflections
Radiation source: fine-focus sealed tube	11851 reflections with *I* > 2σ(*I*)
graphite	*R*_int_ = 0.047
phi and ω scans	θ_max_ = 27.7°, θ_min_ = 1.8°
Absorption correction: multi-scan (*SADABS*; Sheldrick, 1996)	*h* = −15→15
*T*_min_ = 0.733, *T*_max_ = 0.941	*k* = −21→21
49293 measured reflections	*l* = −24→24

### Refinement

**Table:**

Refinement on *F*^2^	Primary atom site location: structure-invariant direct methods
Least-squares matrix: full	Secondary atom site location: difference Fourier map
*R*[*F*^2^ > 2σ(*F*^2^)] = 0.029	Hydrogen site location: inferred from neighbouring sites
*wR*(*F*^2^) = 0.070	H atoms treated by a mixture of independent and constrained refinement
*S* = 1.02	*w* = 1/[σ^2^(*F*_o_^2^) + (0.0236*P*)^2^ + 0.993*P*] where *P* = (*F*_o_^2^ + 2*F*_c_^2^)/3
14715 reflections	(Δ/σ)_max_ = 0.002
875 parameters	Δρ_max_ = 0.66 e Å^−^^3^
9 restraints	Δρ_min_ = −0.72 e Å^−^^3^

### Special details

**Table:**

Geometry. All e.s.d.'s (except the e.s.d. in the dihedral angle between two l.s. planes) are estimated using the full covariance matrix. The cell e.s.d.'s are taken into account individually in the estimation of e.s.d.'s in distances, angles and torsion angles; correlations between e.s.d.'s in cell parameters are only used when they are defined by crystal symmetry. An approximate (isotropic) treatment of cell e.s.d.'s is used for estimating e.s.d.'s involving l.s. planes.
Refinement. Refinement of *F*^2^ against ALL reflections. The weighted *R*-factor *wR* and goodness of fit *S* are based on *F*^2^, conventional *R*-factors *R* are based on *F*, with *F* set to zero for negative *F*^2^. The threshold expression of *F*^2^ > σ(*F*^2^) is used only for calculating *R*-factors(gt) *etc*. and is not relevant to the choice of reflections for refinement. *R*-factors based on *F*^2^ are statistically about twice as large as those based on *F*, and *R*- factors based on ALL data will be even larger.

### Fractional atomic coordinates and isotropic or equivalent isotropic displacement parameters (Å^2^)

**Table:**

	*x*	*y*	*z*	*U*_iso_*/*U*_eq_	
Sm1	0.271174 (13)	0.364512 (8)	0.283786 (7)	0.02579 (4)	
Sm2	0.131899 (13)	0.206492 (8)	0.195381 (7)	0.02577 (4)	
N1	0.3136 (2)	0.51130 (14)	0.28289 (14)	0.0359 (6)	
N2	0.3976 (3)	0.92617 (19)	0.2162 (2)	0.0667 (10)	
N3	0.0840 (2)	0.06204 (14)	0.18856 (13)	0.0337 (5)	
N4	0.0282 (4)	−0.36185 (19)	0.2326 (2)	0.0683 (10)	
C1	0.2248 (3)	0.51101 (19)	0.51768 (16)	0.0363 (7)	
C2	0.3403 (3)	0.5160 (2)	0.47296 (17)	0.0458 (8)	
H2A	0.3962	0.4689	0.4433	0.055*	
C3	0.3750 (3)	0.5896 (2)	0.47116 (19)	0.0505 (8)	
H3A	0.4533	0.5918	0.4404	0.061*	
C4	0.2940 (3)	0.6592 (2)	0.51481 (19)	0.0449 (8)	
C5	0.1799 (3)	0.6549 (2)	0.56137 (19)	0.0482 (8)	
H5A	0.1261	0.7013	0.5926	0.058*	
C6	0.1449 (3)	0.5823 (2)	0.56210 (18)	0.0445 (8)	
H6A	0.0664	0.5808	0.5929	0.053*	
C7	0.1855 (3)	0.4307 (2)	0.52126 (16)	0.0429 (8)	
H7A	0.2316	0.3855	0.5497	0.052*	
H7B	0.0967	0.4440	0.5481	0.052*	
C8	0.2096 (3)	0.39816 (19)	0.44318 (16)	0.0355 (7)	
C9	0.6232 (3)	0.08807 (18)	0.30336 (18)	0.0378 (7)	
C10	0.6052 (3)	0.0570 (2)	0.37691 (18)	0.0492 (8)	
H10A	0.5938	0.0937	0.4154	0.059*	
C11	0.6033 (3)	−0.0276 (2)	0.39568 (18)	0.0494 (8)	
H11A	0.5911	−0.0472	0.4459	0.059*	
C12	0.6199 (3)	−0.08171 (18)	0.33913 (17)	0.0394 (7)	
C13	0.6364 (3)	−0.0518 (2)	0.26524 (17)	0.0438 (8)	
H13A	0.6459	−0.0882	0.2270	0.053*	
C14	0.6389 (3)	0.03153 (19)	0.24766 (17)	0.0432 (7)	
H14A	0.6515	0.0506	0.1973	0.052*	
C15	0.6269 (3)	0.17940 (19)	0.2834 (2)	0.0478 (8)	
H15A	0.6939	0.1783	0.2367	0.057*	
H15B	0.6475	0.2005	0.3233	0.057*	
C16	0.5063 (3)	0.24269 (17)	0.27316 (16)	0.0331 (6)	
C17	0.2720 (3)	0.4547 (2)	0.01289 (16)	0.0424 (8)	
C18	0.1461 (4)	0.5016 (2)	0.02779 (17)	0.0497 (9)	
H18A	0.0861	0.4747	0.0537	0.060*	
C19	0.1070 (3)	0.5873 (2)	0.00524 (17)	0.0453 (8)	
H19A	0.0218	0.6177	0.0158	0.054*	
C20	0.1963 (3)	0.62750 (19)	−0.03339 (17)	0.0405 (7)	
C21	0.3219 (3)	0.58206 (19)	−0.04820 (17)	0.0415 (7)	
H21A	0.3820	0.6090	−0.0737	0.050*	
C22	0.3589 (3)	0.4964 (2)	−0.02525 (17)	0.0434 (7)	
H22A	0.4442	0.4662	−0.0357	0.052*	
C23	0.3132 (4)	0.3613 (2)	0.03920 (17)	0.0587 (10)	
H23A	0.2587	0.3316	0.0315	0.070*	
H23B	0.3981	0.3328	0.0085	0.070*	
C24	0.3094 (3)	0.35389 (17)	0.12169 (15)	0.0330 (6)	
C25	0.1416 (3)	0.11314 (19)	0.47589 (16)	0.0392 (7)	
C26	0.0393 (3)	0.1063 (2)	0.53593 (17)	0.0434 (7)	
H26A	−0.0293	0.1554	0.5521	0.052*	
C27	0.0378 (3)	0.0273 (2)	0.57213 (17)	0.0421 (7)	
H27A	−0.0312	0.0238	0.6125	0.051*	
C28	0.1382 (3)	−0.04551 (19)	0.54821 (16)	0.0359 (7)	
C29	0.2418 (3)	−0.0397 (2)	0.48932 (17)	0.0437 (7)	
H29A	0.3106	−0.0888	0.4735	0.052*	
C30	0.2428 (3)	0.0393 (2)	0.45411 (17)	0.0436 (8)	
H30A	0.3131	0.0429	0.4148	0.052*	
C31	0.1430 (4)	0.1978 (2)	0.43418 (17)	0.0461 (8)	
H31A	0.2238	0.2058	0.4274	0.055*	
H31B	0.0791	0.2446	0.4657	0.055*	
C32	0.1204 (3)	0.20483 (17)	0.35761 (15)	0.0311 (6)	
C33	−0.2564 (3)	0.48057 (17)	0.24478 (16)	0.0322 (6)	
C34	−0.2139 (3)	0.51863 (19)	0.17454 (17)	0.0400 (7)	
H34A	−0.1767	0.4845	0.1313	0.048*	
C35	−0.2258 (3)	0.60528 (19)	0.16781 (17)	0.0412 (7)	
H35A	−0.1963	0.6290	0.1203	0.049*	
C36	−0.2815 (3)	0.65783 (18)	0.23124 (17)	0.0390 (7)	
C37	−0.3244 (3)	0.62157 (19)	0.30141 (17)	0.0431 (7)	
H37A	−0.3622	0.6560	0.3445	0.052*	
C38	−0.3109 (3)	0.53371 (19)	0.30752 (17)	0.0396 (7)	
H38A	−0.3394	0.5099	0.3552	0.048*	
C39	−0.2442 (3)	0.38587 (17)	0.25117 (18)	0.0385 (7)	
H39A	−0.2896	0.3738	0.3026	0.046*	
H39B	−0.2848	0.3736	0.2171	0.046*	
C40	−0.1118 (3)	0.32472 (17)	0.23337 (15)	0.0309 (6)	
C41	0.2359 (3)	0.05195 (19)	−0.04822 (16)	0.0395 (7)	
C42	0.3536 (3)	0.0011 (2)	−0.04455 (16)	0.0430 (7)	
H42A	0.4101	0.0278	−0.0405	0.052*	
C43	0.3896 (3)	−0.0878 (2)	−0.04677 (17)	0.0458 (8)	
H43A	0.4701	−0.1206	−0.0452	0.055*	
C44	0.3057 (3)	−0.1283 (2)	−0.05126 (18)	0.0483 (8)	
C45	0.1879 (3)	−0.0793 (2)	−0.05580 (19)	0.0506 (8)	
H45A	0.1316	−0.1062	−0.0598	0.061*	
C46	0.1538 (3)	0.0102 (2)	−0.05431 (17)	0.0466 (8)	
H46A	0.0742	0.0431	−0.0575	0.056*	
C47	0.1936 (3)	0.1500 (2)	−0.04211 (17)	0.0464 (8)	
H47A	0.2564	0.1724	−0.0781	0.056*	
H47B	0.1156	0.1742	−0.0556	0.056*	
C48	0.1739 (3)	0.17845 (18)	0.03700 (16)	0.0358 (7)	
C49	0.2317 (3)	0.58065 (18)	0.32245 (17)	0.0385 (7)	
H49A	0.1590	0.5745	0.3578	0.046*	
C50	0.2495 (3)	0.66107 (18)	0.31352 (17)	0.0404 (7)	
H50A	0.1897	0.7075	0.3426	0.048*	
C51	0.3563 (3)	0.67260 (18)	0.26136 (17)	0.0377 (7)	
C52	0.4429 (3)	0.60032 (19)	0.22209 (19)	0.0455 (8)	
H52A	0.5175	0.6044	0.1874	0.055*	
C53	0.4187 (3)	0.52211 (19)	0.23437 (19)	0.0435 (8)	
H53A	0.4787	0.4742	0.2074	0.052*	
C54	0.4496 (4)	0.8649 (2)	0.1638 (3)	0.0724 (13)	
H54A	0.4949	0.8787	0.1158	0.087*	
C55	0.4407 (3)	0.7819 (2)	0.1761 (2)	0.0588 (10)	
H55A	0.4788	0.7417	0.1371	0.071*	
C56	0.3751 (3)	0.75940 (19)	0.24675 (19)	0.0427 (8)	
C57	0.3238 (4)	0.8221 (2)	0.3018 (2)	0.0613 (10)	
H57A	0.2810	0.8096	0.3511	0.074*	
C58	0.3363 (5)	0.9036 (2)	0.2835 (3)	0.0726 (12)	
H58A	0.2988	0.9452	0.3214	0.087*	
C59	0.1697 (3)	−0.00447 (18)	0.14641 (17)	0.0406 (7)	
H59A	0.2428	0.0042	0.1136	0.049*	
C60	0.1549 (3)	−0.08558 (18)	0.14936 (17)	0.0420 (7)	
H60A	0.2165	−0.1294	0.1180	0.050*	
C61	0.0493 (3)	−0.10176 (18)	0.19860 (17)	0.0376 (7)	
C62	−0.0413 (3)	−0.03205 (19)	0.24020 (18)	0.0417 (7)	
H62A	−0.1156	−0.0390	0.2729	0.050*	
C63	−0.0217 (3)	0.04762 (18)	0.23331 (17)	0.0390 (7)	
H63A	−0.0850	0.0936	0.2611	0.047*	
C64	0.1071 (5)	−0.3394 (2)	0.1717 (2)	0.0729 (13)	
H64A	0.1613	−0.3824	0.1370	0.088*	
C65	0.1141 (4)	−0.2559 (2)	0.1565 (2)	0.0635 (11)	
H65A	0.1694	−0.2436	0.1116	0.076*	
C66	0.0382 (3)	−0.19087 (19)	0.20833 (19)	0.0445 (8)	
C67	−0.0452 (3)	−0.2136 (2)	0.2724 (2)	0.0622 (11)	
H67A	−0.0988	−0.1725	0.3090	0.075*	
C68	−0.0474 (4)	−0.2986 (2)	0.2811 (3)	0.0751 (14)	
H68A	−0.1056	−0.3124	0.3236	0.090*	
O1W	0.0812 (2)	0.45996 (13)	0.25489 (13)	0.0402 (5)	
O1	0.3063 (2)	0.33572 (14)	0.41483 (12)	0.0464 (5)	
O2W	0.3290 (2)	0.11013 (13)	0.21451 (13)	0.0413 (5)	
O2	0.13551 (19)	0.43506 (13)	0.40444 (11)	0.0393 (5)	
O3	0.3309 (2)	0.73187 (16)	0.51131 (16)	0.0669 (7)	
H3B	0.2729	0.7703	0.5372	0.057 (12)*	
O3W	0.5581 (3)	0.76686 (17)	0.49096 (15)	0.0579 (6)	
O4	0.49742 (19)	0.32158 (12)	0.26433 (13)	0.0470 (6)	
O5	0.41478 (19)	0.21718 (12)	0.27608 (12)	0.0400 (5)	
O6	0.6210 (3)	−0.16638 (13)	0.35286 (12)	0.0596 (7)	
H6B	0.5940	−0.1742	0.3988	0.089*	
O7	0.3524 (2)	0.40008 (14)	0.14780 (11)	0.0460 (6)	
O8	0.25816 (18)	0.30140 (11)	0.16494 (10)	0.0319 (4)	
O9	0.1635 (2)	0.71240 (14)	−0.05719 (14)	0.0560 (6)	
H9A	0.0928	0.7265	−0.0624	0.084*	
O10	0.14230 (18)	0.26764 (11)	0.31403 (10)	0.0308 (4)	
O11	0.0819 (2)	0.15184 (12)	0.33561 (10)	0.0379 (5)	
O12	0.1417 (2)	−0.12554 (13)	0.58163 (12)	0.0468 (5)	
H12A	0.0699	−0.1259	0.6043	0.070*	
O13	−0.01795 (18)	0.35213 (12)	0.20644 (12)	0.0396 (5)	
O14	−0.09509 (19)	0.24475 (12)	0.24404 (12)	0.0436 (5)	
O15	−0.2918 (2)	0.74357 (13)	0.22099 (13)	0.0563 (6)	
H15C	−0.3183	0.7671	0.2626	0.084*	
O16	0.2617 (2)	0.15313 (13)	0.06689 (11)	0.0410 (5)	
O17	0.0674 (2)	0.22729 (14)	0.07417 (12)	0.0479 (5)	
O18	0.3448 (3)	−0.21694 (15)	−0.05145 (16)	0.0742 (8)	
H18B	0.2885	−0.2341	−0.0549	0.111*	
H2WA	0.377 (4)	0.125 (3)	0.231 (3)	0.111*	
H2WB	0.362 (4)	0.0568 (13)	0.208 (3)	0.111*	
H3WB	0.497 (3)	0.748 (3)	0.495 (3)	0.111*	
H1WA	0.043 (4)	0.436 (2)	0.239 (3)	0.111*	
H1WB	0.048 (4)	0.5124 (12)	0.249 (3)	0.111*	
H3WA	0.594 (4)	0.734 (3)	0.521 (2)	0.111*	

### Atomic displacement parameters (Å^2^)

**Table:**

	*U*^11^	*U*^22^	*U*^33^	*U*^12^	*U*^13^	*U*^23^
Sm1	0.02854 (8)	0.01798 (7)	0.03435 (8)	−0.00970 (6)	−0.01220 (6)	0.00222 (5)
Sm2	0.02720 (8)	0.01720 (7)	0.03501 (8)	−0.00788 (6)	−0.01100 (6)	0.00011 (5)
N1	0.0358 (15)	0.0245 (12)	0.0504 (15)	−0.0115 (11)	−0.0146 (12)	−0.0006 (10)
N2	0.065 (2)	0.0320 (16)	0.121 (3)	−0.0237 (16)	−0.049 (2)	0.0175 (18)
N3	0.0388 (15)	0.0216 (11)	0.0433 (14)	−0.0100 (10)	−0.0154 (11)	0.0014 (10)
N4	0.084 (3)	0.0323 (17)	0.119 (3)	−0.0256 (17)	−0.070 (2)	0.0171 (18)
C1	0.0403 (18)	0.0384 (16)	0.0334 (15)	−0.0138 (14)	−0.0124 (13)	−0.0034 (12)
C2	0.0385 (19)	0.0460 (19)	0.0467 (18)	−0.0089 (15)	−0.0036 (15)	−0.0133 (14)
C3	0.0343 (18)	0.053 (2)	0.060 (2)	−0.0180 (16)	0.0001 (16)	−0.0114 (16)
C4	0.0403 (19)	0.0419 (18)	0.055 (2)	−0.0162 (15)	−0.0129 (16)	−0.0037 (14)
C5	0.0385 (19)	0.0425 (18)	0.059 (2)	−0.0096 (15)	−0.0063 (16)	−0.0140 (15)
C6	0.0358 (18)	0.0426 (18)	0.0514 (19)	−0.0124 (15)	−0.0034 (15)	−0.0115 (14)
C7	0.056 (2)	0.0414 (18)	0.0351 (16)	−0.0220 (16)	−0.0108 (15)	0.0002 (13)
C8	0.0425 (19)	0.0340 (16)	0.0354 (15)	−0.0216 (14)	−0.0093 (14)	0.0027 (12)
C9	0.0284 (16)	0.0280 (15)	0.060 (2)	−0.0047 (12)	−0.0215 (14)	−0.0002 (13)
C10	0.067 (2)	0.0310 (17)	0.0507 (19)	−0.0045 (16)	−0.0273 (18)	−0.0096 (14)
C11	0.069 (2)	0.0375 (18)	0.0395 (17)	−0.0109 (16)	−0.0179 (17)	−0.0032 (13)
C12	0.0428 (19)	0.0300 (15)	0.0434 (17)	−0.0105 (13)	−0.0105 (14)	−0.0006 (12)
C13	0.056 (2)	0.0346 (17)	0.0419 (17)	−0.0125 (15)	−0.0155 (15)	−0.0059 (13)
C14	0.051 (2)	0.0368 (17)	0.0403 (17)	−0.0120 (15)	−0.0145 (15)	0.0047 (13)
C15	0.0329 (18)	0.0280 (16)	0.088 (2)	−0.0069 (13)	−0.0296 (17)	0.0056 (15)
C16	0.0306 (16)	0.0269 (14)	0.0416 (16)	−0.0067 (12)	−0.0124 (13)	−0.0004 (11)
C17	0.066 (2)	0.0394 (17)	0.0331 (16)	−0.0272 (17)	−0.0205 (15)	0.0043 (12)
C18	0.067 (3)	0.063 (2)	0.0339 (16)	−0.045 (2)	−0.0108 (16)	0.0033 (15)
C19	0.0409 (19)	0.056 (2)	0.0424 (18)	−0.0187 (16)	−0.0106 (15)	−0.0078 (15)
C20	0.053 (2)	0.0359 (16)	0.0438 (17)	−0.0191 (15)	−0.0251 (15)	0.0029 (13)
C21	0.046 (2)	0.0366 (17)	0.0500 (18)	−0.0219 (15)	−0.0177 (15)	0.0086 (13)
C22	0.051 (2)	0.0382 (17)	0.0470 (18)	−0.0170 (15)	−0.0214 (16)	0.0045 (13)
C23	0.112 (3)	0.0389 (18)	0.0380 (18)	−0.037 (2)	−0.0281 (19)	0.0078 (14)
C24	0.0423 (18)	0.0254 (14)	0.0322 (14)	−0.0139 (13)	−0.0086 (13)	0.0006 (11)
C25	0.058 (2)	0.0380 (17)	0.0354 (16)	−0.0256 (16)	−0.0236 (15)	0.0076 (12)
C26	0.052 (2)	0.0332 (16)	0.0448 (18)	−0.0104 (15)	−0.0177 (16)	−0.0001 (13)
C27	0.0437 (19)	0.0457 (18)	0.0366 (16)	−0.0181 (15)	−0.0084 (14)	0.0049 (13)
C28	0.0446 (19)	0.0354 (16)	0.0358 (15)	−0.0204 (14)	−0.0179 (14)	0.0096 (12)
C29	0.043 (2)	0.0442 (18)	0.0441 (18)	−0.0155 (15)	−0.0135 (15)	0.0063 (14)
C30	0.047 (2)	0.051 (2)	0.0387 (17)	−0.0268 (17)	−0.0119 (15)	0.0109 (14)
C31	0.072 (2)	0.0375 (17)	0.0451 (18)	−0.0312 (17)	−0.0274 (17)	0.0092 (13)
C32	0.0301 (16)	0.0266 (14)	0.0387 (15)	−0.0133 (12)	−0.0089 (12)	0.0021 (11)
C33	0.0274 (15)	0.0256 (14)	0.0424 (16)	−0.0039 (11)	−0.0123 (13)	−0.0026 (11)
C34	0.0435 (19)	0.0350 (16)	0.0377 (16)	−0.0063 (14)	−0.0088 (14)	−0.0122 (12)
C35	0.047 (2)	0.0363 (17)	0.0385 (16)	−0.0137 (14)	−0.0100 (14)	0.0015 (13)
C36	0.0421 (19)	0.0273 (15)	0.0491 (18)	−0.0069 (13)	−0.0192 (15)	−0.0014 (12)
C37	0.052 (2)	0.0341 (16)	0.0404 (17)	−0.0069 (15)	−0.0140 (15)	−0.0077 (13)
C38	0.0403 (18)	0.0344 (16)	0.0390 (16)	−0.0077 (13)	−0.0087 (14)	0.0001 (12)
C39	0.0309 (17)	0.0268 (15)	0.0569 (19)	−0.0050 (12)	−0.0144 (14)	−0.0035 (13)
C40	0.0306 (16)	0.0234 (13)	0.0403 (15)	−0.0060 (12)	−0.0138 (13)	−0.0036 (11)
C41	0.050 (2)	0.0382 (17)	0.0303 (15)	−0.0157 (15)	−0.0087 (14)	−0.0027 (12)
C42	0.048 (2)	0.0441 (18)	0.0407 (17)	−0.0219 (16)	−0.0106 (15)	−0.0005 (13)
C43	0.048 (2)	0.0385 (18)	0.0484 (19)	−0.0121 (15)	−0.0103 (15)	−0.0027 (14)
C44	0.059 (2)	0.0353 (17)	0.0499 (19)	−0.0194 (16)	−0.0095 (17)	−0.0024 (14)
C45	0.053 (2)	0.049 (2)	0.057 (2)	−0.0267 (18)	−0.0118 (17)	−0.0055 (16)
C46	0.043 (2)	0.049 (2)	0.0451 (18)	−0.0098 (16)	−0.0112 (15)	−0.0086 (14)
C47	0.062 (2)	0.0369 (17)	0.0376 (17)	−0.0106 (16)	−0.0155 (16)	0.0003 (13)
C48	0.050 (2)	0.0246 (14)	0.0339 (15)	−0.0152 (14)	−0.0098 (14)	0.0010 (11)
C49	0.0425 (19)	0.0296 (15)	0.0442 (17)	−0.0142 (14)	−0.0111 (14)	0.0019 (12)
C50	0.0465 (19)	0.0244 (15)	0.0516 (18)	−0.0103 (13)	−0.0165 (15)	−0.0014 (12)
C51	0.0402 (18)	0.0245 (14)	0.0552 (18)	−0.0129 (13)	−0.0222 (15)	0.0044 (12)
C52	0.0345 (18)	0.0329 (16)	0.070 (2)	−0.0153 (14)	−0.0117 (16)	0.0025 (15)
C53	0.0365 (18)	0.0257 (15)	0.067 (2)	−0.0103 (13)	−0.0112 (16)	−0.0048 (14)
C54	0.054 (3)	0.041 (2)	0.114 (3)	−0.0220 (19)	−0.013 (2)	0.023 (2)
C55	0.049 (2)	0.0320 (18)	0.085 (3)	−0.0160 (16)	−0.0029 (19)	0.0024 (17)
C56	0.0408 (19)	0.0270 (15)	0.069 (2)	−0.0149 (14)	−0.0258 (16)	0.0054 (14)
C57	0.094 (3)	0.0378 (19)	0.065 (2)	−0.031 (2)	−0.033 (2)	0.0052 (16)
C58	0.112 (4)	0.039 (2)	0.089 (3)	−0.034 (2)	−0.049 (3)	0.0000 (19)
C59	0.0458 (19)	0.0291 (15)	0.0432 (17)	−0.0120 (14)	−0.0086 (15)	0.0028 (12)
C60	0.051 (2)	0.0242 (15)	0.0479 (18)	−0.0088 (14)	−0.0123 (15)	−0.0038 (12)
C61	0.0477 (19)	0.0276 (15)	0.0493 (17)	−0.0142 (14)	−0.0303 (15)	0.0072 (12)
C62	0.0391 (18)	0.0327 (16)	0.0574 (19)	−0.0172 (14)	−0.0145 (15)	0.0034 (14)
C63	0.0381 (18)	0.0269 (15)	0.0547 (18)	−0.0122 (13)	−0.0145 (15)	−0.0014 (13)
C64	0.133 (4)	0.035 (2)	0.076 (3)	−0.028 (2)	−0.063 (3)	−0.0010 (18)
C65	0.118 (4)	0.0349 (19)	0.055 (2)	−0.032 (2)	−0.043 (2)	0.0041 (15)
C66	0.056 (2)	0.0276 (15)	0.066 (2)	−0.0162 (15)	−0.0398 (18)	0.0080 (14)
C67	0.045 (2)	0.0343 (18)	0.109 (3)	−0.0162 (16)	−0.026 (2)	0.0130 (19)
C68	0.054 (3)	0.039 (2)	0.145 (4)	−0.0274 (19)	−0.044 (3)	0.030 (2)
O1W	0.0430 (13)	0.0232 (10)	0.0624 (14)	−0.0089 (9)	−0.0288 (11)	0.0028 (9)
O1	0.0521 (15)	0.0388 (12)	0.0462 (12)	−0.0040 (11)	−0.0207 (11)	−0.0047 (10)
O2W	0.0404 (13)	0.0237 (10)	0.0655 (14)	−0.0073 (9)	−0.0257 (11)	−0.0013 (10)
O2	0.0383 (12)	0.0408 (12)	0.0412 (11)	−0.0132 (10)	−0.0135 (10)	−0.0021 (9)
O3	0.0475 (15)	0.0463 (14)	0.105 (2)	−0.0237 (13)	−0.0038 (15)	−0.0185 (14)
O3W	0.0609 (18)	0.0564 (16)	0.0613 (16)	−0.0235 (14)	−0.0260 (13)	0.0190 (12)
O4	0.0360 (12)	0.0236 (10)	0.0820 (16)	−0.0104 (9)	−0.0201 (11)	0.0091 (10)
O5	0.0338 (12)	0.0249 (10)	0.0686 (14)	−0.0079 (9)	−0.0251 (10)	−0.0035 (9)
O6	0.096 (2)	0.0339 (12)	0.0485 (13)	−0.0285 (13)	−0.0118 (13)	−0.0006 (10)
O7	0.0632 (15)	0.0504 (13)	0.0399 (12)	−0.0408 (12)	−0.0145 (11)	0.0078 (9)
O8	0.0365 (11)	0.0243 (10)	0.0378 (10)	−0.0148 (8)	−0.0106 (9)	0.0039 (8)
O9	0.0606 (16)	0.0360 (12)	0.0839 (17)	−0.0159 (11)	−0.0408 (14)	0.0096 (11)
O10	0.0356 (11)	0.0241 (9)	0.0349 (10)	−0.0144 (8)	−0.0096 (8)	0.0049 (7)
O11	0.0527 (14)	0.0350 (11)	0.0373 (11)	−0.0272 (10)	−0.0170 (10)	0.0073 (8)
O12	0.0511 (14)	0.0344 (11)	0.0541 (13)	−0.0201 (10)	−0.0117 (11)	0.0130 (9)
O13	0.0279 (11)	0.0236 (10)	0.0687 (14)	−0.0064 (8)	−0.0167 (10)	−0.0029 (9)
O14	0.0330 (12)	0.0220 (10)	0.0713 (15)	−0.0078 (9)	−0.0105 (10)	0.0022 (9)
O15	0.0844 (19)	0.0294 (12)	0.0591 (14)	−0.0195 (12)	−0.0253 (13)	0.0020 (10)
O16	0.0394 (13)	0.0397 (12)	0.0450 (12)	−0.0109 (10)	−0.0131 (10)	−0.0062 (9)
O17	0.0487 (14)	0.0455 (13)	0.0436 (12)	0.0021 (11)	−0.0204 (11)	−0.0075 (10)
O18	0.077 (2)	0.0375 (14)	0.112 (2)	−0.0190 (14)	−0.0312 (17)	−0.0027 (14)

### Geometric parameters (Å, °)

**Table:**

Sm1—O5	2.4344 (19)	C28—O12	1.374 (3)
Sm1—O4	2.439 (2)	C28—C29	1.382 (4)
Sm1—O1W	2.445 (2)	C29—C30	1.379 (4)
Sm1—O10	2.4396 (16)	C29—H29A	0.9300
Sm1—O2	2.451 (2)	C30—H30A	0.9300
Sm1—O7	2.4718 (19)	C31—C32	1.502 (4)
Sm1—O1	2.546 (2)	C31—H31A	0.9700
Sm1—O8	2.5904 (18)	C31—H31B	0.9700
Sm1—N1	2.598 (2)	C32—O11	1.251 (3)
Sm1—C16	2.792 (3)	C32—O10	1.274 (3)
Sm1—C8	2.876 (3)	C33—C38	1.381 (4)
Sm1—C24	2.902 (3)	C33—C34	1.393 (4)
Sm2—O8	2.3896 (16)	C33—C39	1.499 (4)
Sm2—O14	2.424 (2)	C34—C35	1.370 (4)
Sm2—O13	2.4379 (19)	C34—H34A	0.9300
Sm2—O2W	2.444 (2)	C35—C36	1.385 (4)
Sm2—O16	2.464 (2)	C35—H35A	0.9300
Sm2—O17	2.532 (2)	C36—O15	1.361 (3)
Sm2—O10	2.5572 (18)	C36—C37	1.380 (4)
Sm2—O11	2.5947 (18)	C37—C38	1.385 (4)
Sm2—N3	2.620 (2)	C37—H37A	0.9300
Sm2—C40	2.806 (3)	C38—H38A	0.9300
Sm2—C48	2.874 (3)	C39—C40	1.506 (4)
Sm2—C32	2.954 (3)	C39—H39A	0.9700
N1—C53	1.336 (4)	C39—H39B	0.9700
N1—C49	1.334 (4)	C40—O14	1.257 (3)
N2—C58	1.312 (5)	C40—O13	1.265 (3)
N2—C54	1.326 (5)	C41—C42	1.380 (4)
N3—C63	1.341 (4)	C41—C46	1.386 (4)
N3—C59	1.337 (4)	C41—C47	1.517 (4)
N4—C64	1.320 (5)	C42—C43	1.373 (4)
N4—C68	1.331 (6)	C42—H42A	0.9300
C1—C2	1.378 (4)	C43—C44	1.380 (4)
C1—C6	1.389 (4)	C43—H43A	0.9300
C1—C7	1.514 (4)	C44—O18	1.368 (4)
C2—C3	1.383 (4)	C44—C45	1.378 (5)
C2—H2A	0.9300	C45—C46	1.383 (4)
C3—C4	1.367 (4)	C45—H45A	0.9300
C3—H3A	0.9300	C46—H46A	0.9300
C4—C5	1.372 (4)	C47—C48	1.508 (4)
C4—O3	1.378 (4)	C47—H47A	0.9700
C5—C6	1.372 (4)	C47—H47B	0.9700
C5—H5A	0.9300	C48—O16	1.250 (4)
C6—H6A	0.9300	C48—O17	1.271 (4)
C7—C8	1.503 (4)	C49—C50	1.379 (4)
C7—H7A	0.9700	C49—H49A	0.9300
C7—H7B	0.9700	C50—C51	1.381 (4)
C8—O1	1.264 (4)	C50—H50A	0.9300
C8—O2	1.257 (4)	C51—C52	1.380 (4)
C9—C10	1.373 (4)	C51—C56	1.486 (4)
C9—C14	1.387 (4)	C52—C53	1.376 (4)
C9—C15	1.506 (4)	C52—H52A	0.9300
C10—C11	1.392 (4)	C53—H53A	0.9300
C10—H10A	0.9300	C54—C55	1.380 (4)
C11—C12	1.369 (4)	C54—H54A	0.9300
C11—H11A	0.9300	C55—C56	1.374 (5)
C12—C13	1.374 (4)	C55—H55A	0.9300
C12—O6	1.373 (3)	C56—C57	1.376 (5)
C13—C14	1.369 (4)	C57—C58	1.378 (5)
C13—H13A	0.9300	C57—H57A	0.9300
C14—H14A	0.9300	C58—H58A	0.9300
C15—C16	1.509 (4)	C59—C60	1.383 (4)
C15—H15A	0.9700	C59—H59A	0.9300
C15—H15B	0.9700	C60—C61	1.380 (4)
C16—O4	1.253 (3)	C60—H60A	0.9300
C16—O5	1.260 (3)	C61—C62	1.384 (4)
C17—C22	1.381 (4)	C61—C66	1.488 (4)
C17—C18	1.381 (5)	C62—C63	1.378 (4)
C17—C23	1.514 (4)	C62—H62A	0.9300
C18—C19	1.382 (5)	C63—H63A	0.9300
C18—H18A	0.9300	C64—C65	1.384 (5)
C19—C20	1.387 (4)	C64—H64A	0.9300
C19—H19A	0.9300	C65—C66	1.385 (5)
C20—O9	1.372 (3)	C65—H65A	0.9300
C20—C21	1.373 (4)	C66—C67	1.388 (5)
C21—C22	1.381 (4)	C67—C68	1.385 (5)
C21—H21A	0.9300	C67—H67A	0.9300
C22—H22A	0.9300	C68—H68A	0.9300
C23—C24	1.502 (4)	O1W—H1WA	0.81 (5)
C23—H23A	0.9700	O1W—H1WB	0.826 (18)
C23—H23B	0.9700	O2W—H2WA	0.83 (5)
C24—O7	1.239 (3)	O2W—H2WB	0.834 (18)
C24—O8	1.274 (3)	O3—H3B	0.8200
C25—C30	1.380 (4)	O3W—H3WB	0.85 (4)
C25—C26	1.388 (4)	O3W—H3WA	0.84 (4)
C25—C31	1.507 (4)	O6—H6B	0.8200
C26—C27	1.386 (4)	O9—H9A	0.8200
C26—H26A	0.9300	O12—H12A	0.8200
C27—C28	1.371 (4)	O15—H15C	0.8200
C27—H27A	0.9300	O18—H18B	0.8200
			
O5—Sm1—O4	53.20 (6)	C17—C18—H18A	119.2
O5—Sm1—O1W	145.23 (6)	C18—C19—C20	119.3 (3)
O4—Sm1—O1W	150.49 (7)	C18—C19—H19A	120.3
O5—Sm1—O10	73.25 (6)	C20—C19—H19A	120.3
O4—Sm1—O10	126.11 (6)	O9—C20—C21	118.2 (3)
O1W—Sm1—O10	79.74 (6)	O9—C20—C19	122.0 (3)
O5—Sm1—O2	123.26 (7)	C21—C20—C19	119.8 (3)
O4—Sm1—O2	116.84 (7)	C20—C21—C22	119.9 (3)
O1W—Sm1—O2	74.90 (7)	C20—C21—H21A	120.0
O10—Sm1—O2	85.59 (6)	C22—C21—H21A	120.0
O5—Sm1—O7	95.18 (7)	C17—C22—C21	121.4 (3)
O4—Sm1—O7	77.29 (7)	C17—C22—H22A	119.3
O1W—Sm1—O7	77.80 (8)	C21—C22—H22A	119.3
O10—Sm1—O7	116.92 (6)	C24—C23—C17	112.3 (3)
O2—Sm1—O7	140.51 (7)	C24—C23—H23A	109.2
O5—Sm1—O1	75.97 (7)	C17—C23—H23A	109.2
O4—Sm1—O1	72.89 (8)	C24—C23—H23B	109.2
O1W—Sm1—O1	126.25 (8)	C17—C23—H23B	109.2
O10—Sm1—O1	90.25 (6)	H23A—C23—H23B	107.9
O2—Sm1—O1	51.61 (7)	O7—C24—O8	120.4 (2)
O7—Sm1—O1	148.06 (7)	O7—C24—C23	120.4 (2)
O5—Sm1—O8	75.80 (6)	O8—C24—C23	119.2 (3)
O4—Sm1—O8	102.16 (7)	O7—C24—Sm1	57.66 (14)
O1W—Sm1—O8	73.48 (7)	O8—C24—Sm1	63.20 (14)
O10—Sm1—O8	66.26 (6)	C23—C24—Sm1	171.2 (2)
O2—Sm1—O8	140.65 (6)	C30—C25—C26	118.0 (3)
O7—Sm1—O8	50.96 (6)	C30—C25—C31	120.3 (3)
O1—Sm1—O8	147.46 (6)	C26—C25—C31	121.7 (3)
O5—Sm1—N1	129.77 (7)	C27—C26—C25	121.0 (3)
O4—Sm1—N1	76.68 (7)	C27—C26—H26A	119.5
O1W—Sm1—N1	81.72 (7)	C25—C26—H26A	119.5
O10—Sm1—N1	155.06 (7)	C28—C27—C26	119.8 (3)
O2—Sm1—N1	73.54 (7)	C28—C27—H27A	120.1
O7—Sm1—N1	74.76 (7)	C26—C27—H27A	120.1
O1—Sm1—N1	87.38 (7)	C27—C28—O12	122.4 (3)
O8—Sm1—N1	123.48 (6)	C27—C28—C29	120.0 (3)
O5—Sm1—C16	26.79 (7)	O12—C28—C29	117.6 (3)
O4—Sm1—C16	26.63 (7)	C30—C29—C28	119.7 (3)
O1W—Sm1—C16	163.53 (8)	C30—C29—H29A	120.1
O10—Sm1—C16	99.49 (7)	C28—C29—H29A	120.1
O2—Sm1—C16	121.54 (8)	C29—C30—C25	121.4 (3)
O7—Sm1—C16	88.13 (8)	C29—C30—H30A	119.3
O1—Sm1—C16	70.06 (8)	C25—C30—H30A	119.3
O8—Sm1—C16	91.05 (7)	C25—C31—C32	114.9 (2)
N1—Sm1—C16	102.98 (8)	C25—C31—H31A	108.5
O5—Sm1—C8	100.86 (8)	C32—C31—H31A	108.5
O4—Sm1—C8	93.91 (8)	C25—C31—H31B	108.5
O1W—Sm1—C8	100.65 (9)	C32—C31—H31B	108.5
O10—Sm1—C8	90.08 (7)	H31A—C31—H31B	107.5
O2—Sm1—C8	25.75 (8)	O11—C32—O10	119.6 (2)
O7—Sm1—C8	151.75 (7)	O11—C32—C31	122.7 (2)
O1—Sm1—C8	26.07 (8)	O10—C32—C31	117.7 (2)
O8—Sm1—C8	156.18 (6)	O11—C32—Sm2	61.23 (14)
N1—Sm1—C8	77.11 (7)	O10—C32—Sm2	59.60 (13)
C16—Sm1—C8	95.79 (9)	C31—C32—Sm2	168.4 (2)
O5—Sm1—C24	86.95 (8)	C38—C33—C34	117.3 (2)
O4—Sm1—C24	91.04 (8)	C38—C33—C39	121.8 (3)
O1W—Sm1—C24	72.29 (8)	C34—C33—C39	120.9 (3)
O10—Sm1—C24	91.89 (7)	C35—C34—C33	121.4 (3)
O2—Sm1—C24	147.01 (8)	C35—C34—H34A	119.3
O7—Sm1—C24	25.05 (7)	C33—C34—H34A	119.3
O1—Sm1—C24	161.38 (8)	C34—C35—C36	120.5 (3)
O8—Sm1—C24	26.03 (6)	C34—C35—H35A	119.7
N1—Sm1—C24	98.21 (7)	C36—C35—H35A	119.7
C16—Sm1—C24	91.34 (8)	O15—C36—C37	123.0 (3)
C8—Sm1—C24	172.18 (8)	O15—C36—C35	117.9 (3)
O8—Sm2—O14	127.89 (6)	C37—C36—C35	119.0 (3)
O8—Sm2—O13	75.13 (6)	C36—C37—C38	119.9 (3)
O14—Sm2—O13	53.27 (6)	C36—C37—H37A	120.1
O8—Sm2—O2W	78.76 (7)	C38—C37—H37A	120.1
O14—Sm2—O2W	143.94 (7)	C33—C38—C37	121.8 (3)
O13—Sm2—O2W	147.65 (7)	C33—C38—H38A	119.1
O8—Sm2—O16	81.26 (6)	C37—C38—H38A	119.1
O14—Sm2—O16	126.48 (7)	C40—C39—C33	115.7 (2)
O13—Sm2—O16	117.96 (7)	C40—C39—H39A	108.4
O2W—Sm2—O16	75.82 (8)	C33—C39—H39A	108.4
O8—Sm2—O17	98.20 (7)	C40—C39—H39B	108.4
O14—Sm2—O17	77.89 (7)	C33—C39—H39B	108.4
O13—Sm2—O17	76.05 (7)	H39A—C39—H39B	107.4
O2W—Sm2—O17	126.91 (7)	O14—C40—O13	119.7 (3)
O16—Sm2—O17	51.67 (7)	O14—C40—C39	119.0 (2)
O8—Sm2—O10	67.51 (6)	O13—C40—C39	121.4 (2)
O14—Sm2—O10	90.92 (7)	O14—C40—Sm2	59.52 (14)
O13—Sm2—O10	75.70 (6)	O13—C40—Sm2	60.16 (14)
O2W—Sm2—O10	76.88 (7)	C39—C40—Sm2	177.20 (19)
O16—Sm2—O10	141.76 (6)	C42—C41—C46	117.7 (3)
O17—Sm2—O10	150.83 (6)	C42—C41—C47	122.1 (3)
O8—Sm2—O11	115.29 (6)	C46—C41—C47	120.2 (3)
O14—Sm2—O11	73.46 (7)	C43—C42—C41	121.9 (3)
O13—Sm2—O11	101.66 (7)	C43—C42—H42A	119.0
O2W—Sm2—O11	72.75 (7)	C41—C42—H42A	119.0
O16—Sm2—O11	140.11 (7)	C44—C43—C42	119.6 (3)
O17—Sm2—O11	144.90 (7)	C44—C43—H43A	120.2
O10—Sm2—O11	50.14 (5)	C42—C43—H43A	120.2
O8—Sm2—N3	155.05 (7)	O18—C44—C43	117.5 (3)
O14—Sm2—N3	75.76 (7)	O18—C44—C45	122.6 (3)
O13—Sm2—N3	126.14 (7)	C43—C44—C45	119.9 (3)
O2W—Sm2—N3	84.29 (7)	C46—C45—C44	119.6 (3)
O16—Sm2—N3	76.90 (7)	C46—C45—H45A	120.2
O17—Sm2—N3	77.54 (7)	C44—C45—H45A	120.2
O10—Sm2—N3	126.09 (6)	C45—C46—C41	121.3 (3)
O11—Sm2—N3	76.13 (6)	C45—C46—H46A	119.4
O8—Sm2—C40	101.68 (7)	C41—C46—H46A	119.4
O14—Sm2—C40	26.53 (7)	C48—C47—C41	112.1 (2)
O13—Sm2—C40	26.75 (7)	C48—C47—H47A	109.2
O2W—Sm2—C40	157.91 (8)	C41—C47—H47A	109.2
O16—Sm2—C40	126.24 (8)	C48—C47—H47B	109.2
O17—Sm2—C40	75.11 (7)	C41—C47—H47B	109.2
O10—Sm2—C40	82.88 (7)	H47A—C47—H47B	107.9
O11—Sm2—C40	87.53 (7)	O16—C48—O17	119.5 (3)
N3—Sm2—C40	100.89 (7)	O16—C48—C47	120.3 (3)
O8—Sm2—C48	91.70 (7)	O17—C48—C47	120.2 (3)
O14—Sm2—C48	101.89 (9)	O16—C48—Sm2	58.51 (15)
O13—Sm2—C48	98.78 (8)	O17—C48—Sm2	61.67 (15)
O2W—Sm2—C48	100.77 (9)	C47—C48—Sm2	171.01 (19)
O16—Sm2—C48	25.63 (8)	N1—C49—C50	123.3 (3)
O17—Sm2—C48	26.22 (8)	N1—C49—H49A	118.4
O10—Sm2—C48	159.18 (7)	C50—C49—H49A	118.4
O11—Sm2—C48	149.49 (7)	C49—C50—C51	119.9 (3)
N3—Sm2—C48	73.54 (7)	C49—C50—H50A	120.1
C40—Sm2—C48	101.29 (9)	C51—C50—H50A	120.1
O8—Sm2—C32	90.76 (6)	C50—C51—C52	116.9 (2)
O14—Sm2—C32	84.15 (8)	C50—C51—C56	121.4 (3)
O13—Sm2—C32	91.20 (7)	C52—C51—C56	121.7 (3)
O2W—Sm2—C32	70.24 (8)	C53—C52—C51	119.8 (3)
O16—Sm2—C32	146.03 (8)	C53—C52—H52A	120.1
O17—Sm2—C32	161.85 (8)	C51—C52—H52A	120.1
O10—Sm2—C32	25.46 (6)	N1—C53—C52	123.5 (3)
O11—Sm2—C32	25.00 (6)	N1—C53—H53A	118.2
N3—Sm2—C32	100.64 (7)	C52—C53—H53A	118.2
C40—Sm2—C32	87.67 (8)	N2—C54—C55	124.2 (4)
C48—Sm2—C32	170.02 (8)	N2—C54—H54A	117.9
C53—N1—C49	116.6 (2)	C55—C54—H54A	117.9
C53—N1—Sm1	118.68 (18)	C54—C55—C56	119.2 (4)
C49—N1—Sm1	124.39 (18)	C54—C55—H55A	120.4
C58—N2—C54	115.9 (3)	C56—C55—H55A	120.4
C63—N3—C59	116.5 (2)	C57—C56—C55	116.8 (3)
C63—N3—Sm2	121.64 (18)	C57—C56—C51	122.0 (3)
C59—N3—Sm2	121.42 (18)	C55—C56—C51	121.1 (3)
C64—N4—C68	116.2 (3)	C56—C57—C58	119.6 (4)
C2—C1—C6	117.5 (3)	C56—C57—H57A	120.2
C2—C1—C7	122.4 (3)	C58—C57—H57A	120.2
C6—C1—C7	120.1 (3)	N2—C58—C57	124.2 (4)
C1—C2—C3	121.5 (3)	N2—C58—H58A	117.9
C1—C2—H2A	119.3	C57—C58—H58A	117.9
C3—C2—H2A	119.3	N3—C59—C60	123.1 (3)
C4—C3—C2	119.8 (3)	N3—C59—H59A	118.4
C4—C3—H3A	120.1	C60—C59—H59A	118.4
C2—C3—H3A	120.1	C59—C60—C61	120.3 (3)
C3—C4—C5	119.7 (3)	C59—C60—H60A	119.9
C3—C4—O3	118.5 (3)	C61—C60—H60A	119.9
C5—C4—O3	121.8 (3)	C60—C61—C62	116.4 (2)
C4—C5—C6	120.3 (3)	C60—C61—C66	120.8 (3)
C4—C5—H5A	119.9	C62—C61—C66	122.7 (3)
C6—C5—H5A	119.9	C63—C62—C61	120.2 (3)
C5—C6—C1	121.2 (3)	C63—C62—H62A	119.9
C5—C6—H6A	119.4	C61—C62—H62A	119.9
C1—C6—H6A	119.4	N3—C63—C62	123.2 (3)
C8—C7—C1	111.8 (2)	N3—C63—H63A	118.4
C8—C7—H7A	109.3	C62—C63—H63A	118.4
C1—C7—H7A	109.3	N4—C64—C65	124.0 (4)
C8—C7—H7B	109.3	N4—C64—H64A	118.0
C1—C7—H7B	109.3	C65—C64—H64A	118.0
H7A—C7—H7B	107.9	C64—C65—C66	119.7 (4)
O1—C8—O2	119.4 (3)	C64—C65—H65A	120.2
O1—C8—C7	120.7 (3)	C66—C65—H65A	120.2
O2—C8—C7	119.9 (3)	C65—C66—C67	116.8 (3)
O1—C8—Sm1	62.24 (15)	C65—C66—C61	122.2 (3)
O2—C8—Sm1	57.91 (15)	C67—C66—C61	120.9 (3)
C7—C8—Sm1	169.06 (18)	C68—C67—C66	118.9 (4)
C10—C9—C14	117.2 (3)	C68—C67—H67A	120.5
C10—C9—C15	121.7 (3)	C66—C67—H67A	120.5
C14—C9—C15	121.1 (3)	N4—C68—C67	124.4 (4)
C9—C10—C11	122.1 (3)	N4—C68—H68A	117.8
C9—C10—H10A	119.0	C67—C68—H68A	117.8
C11—C10—H10A	119.0	Sm1—O1W—H1WA	116 (3)
C12—C11—C10	119.1 (3)	Sm1—O1W—H1WB	138 (3)
C12—C11—H11A	120.5	H1WA—O1W—H1WB	105 (3)
C10—C11—H11A	120.5	C8—O1—Sm1	91.69 (17)
C13—C12—C11	119.9 (3)	Sm2—O2W—H2WA	125 (3)
C13—C12—O6	117.5 (3)	Sm2—O2W—H2WB	130 (3)
C11—C12—O6	122.6 (3)	H2WA—O2W—H2WB	105 (3)
C12—C13—C14	120.2 (3)	C8—O2—Sm1	96.34 (18)
C12—C13—H13A	119.9	C4—O3—H3B	109.5
C14—C13—H13A	119.9	H3WB—O3W—H3WA	102 (3)
C13—C14—C9	121.5 (3)	C16—O4—Sm1	92.64 (17)
C13—C14—H14A	119.3	C16—O5—Sm1	92.66 (16)
C9—C14—H14A	119.3	C12—O6—H6B	109.5
C9—C15—C16	114.9 (2)	C24—O7—Sm1	97.28 (16)
C9—C15—H15A	108.5	C24—O8—Sm2	153.69 (18)
C16—C15—H15A	108.5	C24—O8—Sm1	90.77 (15)
C9—C15—H15B	108.5	Sm2—O8—Sm1	113.39 (7)
C16—C15—H15B	108.5	C20—O9—H9A	109.5
H15A—C15—H15B	107.5	C32—O10—Sm1	142.67 (17)
O4—C16—O5	120.5 (3)	C32—O10—Sm2	94.93 (15)
O4—C16—C15	118.7 (3)	Sm1—O10—Sm2	112.84 (6)
O5—C16—C15	120.7 (2)	C32—O11—Sm2	93.77 (15)
O4—C16—Sm1	60.73 (15)	C28—O12—H12A	109.5
O5—C16—Sm1	60.56 (14)	C40—O13—Sm2	93.09 (15)
C15—C16—Sm1	169.1 (2)	C40—O14—Sm2	93.95 (16)
C22—C17—C18	117.9 (3)	C36—O15—H15C	109.5
C22—C17—C23	121.1 (3)	C48—O16—Sm2	95.86 (18)
C18—C17—C23	121.0 (3)	C48—O17—Sm2	92.11 (18)
C19—C18—C17	121.7 (3)	C44—O18—H18B	109.5
C19—C18—H18A	119.2		
			
O5—Sm1—N1—C53	41.9 (3)	O13—Sm2—C48—O17	−30.02 (17)
O4—Sm1—N1—C53	38.2 (2)	O2W—Sm2—C48—O17	175.88 (16)
O1W—Sm1—N1—C53	−121.6 (2)	O16—Sm2—C48—O17	−170.8 (3)
O10—Sm1—N1—C53	−163.8 (2)	O10—Sm2—C48—O17	−102.7 (3)
O2—Sm1—N1—C53	161.8 (2)	O11—Sm2—C48—O17	101.6 (2)
O7—Sm1—N1—C53	−42.0 (2)	N3—Sm2—C48—O17	95.19 (17)
O1—Sm1—N1—C53	111.2 (2)	C40—Sm2—C48—O17	−2.97 (18)
O8—Sm1—N1—C53	−57.7 (2)	C53—N1—C49—C50	2.0 (5)
C16—Sm1—N1—C53	42.4 (2)	Sm1—N1—C49—C50	−171.2 (2)
C8—Sm1—N1—C53	135.4 (2)	N1—C49—C50—C51	0.1 (5)
C24—Sm1—N1—C53	−50.9 (2)	C49—C50—C51—C52	−2.1 (5)
O5—Sm1—N1—C49	−145.0 (2)	C49—C50—C51—C56	176.2 (3)
O4—Sm1—N1—C49	−148.7 (2)	C50—C51—C52—C53	1.9 (5)
O1W—Sm1—N1—C49	51.5 (2)	C56—C51—C52—C53	−176.5 (3)
O10—Sm1—N1—C49	9.2 (3)	C49—N1—C53—C52	−2.3 (5)
O2—Sm1—N1—C49	−25.1 (2)	Sm1—N1—C53—C52	171.4 (3)
O7—Sm1—N1—C49	131.0 (2)	C51—C52—C53—N1	0.3 (5)
O1—Sm1—N1—C49	−75.7 (2)	C58—N2—C54—C55	1.1 (6)
O8—Sm1—N1—C49	115.4 (2)	N2—C54—C55—C56	−0.5 (6)
C16—Sm1—N1—C49	−144.6 (2)	C54—C55—C56—C57	−1.2 (5)
C8—Sm1—N1—C49	−51.5 (2)	C54—C55—C56—C51	176.2 (3)
C24—Sm1—N1—C49	122.1 (2)	C50—C51—C56—C57	29.0 (5)
O8—Sm2—N3—C63	173.16 (19)	C52—C51—C56—C57	−152.8 (3)
O14—Sm2—N3—C63	−23.8 (2)	C50—C51—C56—C55	−148.3 (3)
O13—Sm2—N3—C63	−42.1 (2)	C52—C51—C56—C55	30.0 (5)
O2W—Sm2—N3—C63	125.9 (2)	C55—C56—C57—C58	2.2 (6)
O16—Sm2—N3—C63	−157.3 (2)	C51—C56—C57—C58	−175.1 (3)
O17—Sm2—N3—C63	−104.2 (2)	C54—N2—C58—C57	0.0 (6)
O10—Sm2—N3—C63	56.8 (2)	C56—C57—C58—N2	−1.7 (7)
O11—Sm2—N3—C63	52.3 (2)	C63—N3—C59—C60	−2.1 (4)
C40—Sm2—N3—C63	−32.3 (2)	Sm2—N3—C59—C60	170.8 (2)
C48—Sm2—N3—C63	−131.0 (2)	N3—C59—C60—C61	−1.5 (5)
C32—Sm2—N3—C63	57.3 (2)	C59—C60—C61—C62	3.5 (4)
O8—Sm2—N3—C59	0.6 (3)	C59—C60—C61—C66	−173.5 (3)
O14—Sm2—N3—C59	163.7 (2)	C60—C61—C62—C63	−2.2 (4)
O13—Sm2—N3—C59	145.3 (2)	C66—C61—C62—C63	174.8 (3)
O2W—Sm2—N3—C59	−46.6 (2)	C59—N3—C63—C62	3.6 (4)
O16—Sm2—N3—C59	30.1 (2)	Sm2—N3—C63—C62	−169.3 (2)
O17—Sm2—N3—C59	83.2 (2)	C61—C62—C63—N3	−1.5 (5)
O10—Sm2—N3—C59	−115.8 (2)	C68—N4—C64—C65	−0.3 (6)
O11—Sm2—N3—C59	−120.2 (2)	N4—C64—C65—C66	2.5 (6)
C40—Sm2—N3—C59	155.1 (2)	C64—C65—C66—C67	−2.4 (5)
C48—Sm2—N3—C59	56.4 (2)	C64—C65—C66—C61	174.5 (3)
C32—Sm2—N3—C59	−115.2 (2)	C60—C61—C66—C65	−17.0 (5)
C6—C1—C2—C3	−1.1 (5)	C62—C61—C66—C65	166.1 (3)
C7—C1—C2—C3	−179.2 (3)	C60—C61—C66—C67	159.7 (3)
C1—C2—C3—C4	0.4 (5)	C62—C61—C66—C67	−17.1 (5)
C2—C3—C4—C5	1.4 (5)	C65—C66—C67—C68	0.4 (5)
C2—C3—C4—O3	−179.5 (3)	C61—C66—C67—C68	−176.5 (3)
C3—C4—C5—C6	−2.5 (5)	C64—N4—C68—C67	−1.9 (6)
O3—C4—C5—C6	178.4 (3)	C66—C67—C68—N4	1.9 (6)
C4—C5—C6—C1	1.8 (5)	O2—C8—O1—Sm1	9.7 (3)
C2—C1—C6—C5	0.0 (5)	C7—C8—O1—Sm1	−167.9 (2)
C7—C1—C6—C5	178.2 (3)	O5—Sm1—O1—C8	−162.32 (17)
C2—C1—C7—C8	−48.6 (4)	O4—Sm1—O1—C8	142.36 (17)
C6—C1—C7—C8	133.4 (3)	O1W—Sm1—O1—C8	−12.25 (19)
C1—C7—C8—O1	99.3 (3)	O10—Sm1—O1—C8	−89.67 (16)
C1—C7—C8—O2	−78.3 (3)	O2—Sm1—O1—C8	−5.50 (15)
C1—C7—C8—Sm1	−3.1 (14)	O7—Sm1—O1—C8	120.68 (18)
O5—Sm1—C8—O1	17.46 (17)	O8—Sm1—O1—C8	−131.82 (16)
O4—Sm1—C8—O1	−35.80 (16)	N1—Sm1—O1—C8	65.50 (16)
O1W—Sm1—C8—O1	169.98 (16)	C16—Sm1—O1—C8	170.32 (18)
O10—Sm1—C8—O1	90.41 (16)	C24—Sm1—O1—C8	173.7 (2)
O2—Sm1—C8—O1	170.0 (3)	O1—C8—O2—Sm1	−10.1 (3)
O7—Sm1—C8—O1	−106.0 (2)	C7—C8—O2—Sm1	167.5 (2)
O8—Sm1—C8—O1	96.9 (2)	O5—Sm1—O2—C8	32.73 (18)
N1—Sm1—C8—O1	−111.17 (17)	O4—Sm1—O2—C8	−29.18 (17)
C16—Sm1—C8—O1	−9.14 (17)	O1W—Sm1—O2—C8	179.93 (17)
O5—Sm1—C8—O2	−152.59 (15)	O10—Sm1—O2—C8	99.38 (16)
O4—Sm1—C8—O2	154.15 (15)	O7—Sm1—O2—C8	−132.26 (16)
O1W—Sm1—C8—O2	−0.07 (16)	O1—Sm1—O2—C8	5.56 (15)
O10—Sm1—C8—O2	−79.64 (16)	O8—Sm1—O2—C8	142.44 (15)
O7—Sm1—C8—O2	83.9 (2)	N1—Sm1—O2—C8	−94.42 (16)
O1—Sm1—C8—O2	−170.0 (3)	C16—Sm1—O2—C8	0.94 (18)
O8—Sm1—C8—O2	−73.2 (2)	C24—Sm1—O2—C8	−173.95 (15)
N1—Sm1—C8—O2	78.78 (16)	O5—C16—O4—Sm1	9.9 (3)
C16—Sm1—C8—O2	−179.19 (16)	C15—C16—O4—Sm1	−167.6 (3)
O5—Sm1—C8—C7	125.7 (12)	O5—Sm1—O4—C16	−5.52 (16)
O4—Sm1—C8—C7	72.5 (12)	O1W—Sm1—O4—C16	−145.07 (18)
O1W—Sm1—C8—C7	−81.8 (12)	O10—Sm1—O4—C16	2.2 (2)
O10—Sm1—C8—C7	−161.3 (12)	O2—Sm1—O4—C16	107.38 (18)
O2—Sm1—C8—C7	−81.7 (12)	O7—Sm1—O4—C16	−112.04 (18)
O7—Sm1—C8—C7	2.3 (13)	O1—Sm1—O4—C16	79.52 (18)
O1—Sm1—C8—C7	108.3 (13)	O8—Sm1—O4—C16	−67.19 (18)
O8—Sm1—C8—C7	−154.9 (11)	N1—Sm1—O4—C16	170.88 (19)
N1—Sm1—C8—C7	−2.9 (12)	C8—Sm1—O4—C16	95.12 (18)
C16—Sm1—C8—C7	99.1 (12)	C24—Sm1—O4—C16	−90.93 (18)
C14—C9—C10—C11	0.4 (5)	O4—C16—O5—Sm1	−10.0 (3)
C15—C9—C10—C11	−179.1 (3)	C15—C16—O5—Sm1	167.5 (3)
C9—C10—C11—C12	−0.1 (5)	O4—Sm1—O5—C16	5.49 (16)
C10—C11—C12—C13	−0.8 (5)	O1W—Sm1—O5—C16	151.41 (17)
C10—C11—C12—O6	179.1 (3)	O10—Sm1—O5—C16	−168.04 (18)
C11—C12—C13—C14	1.3 (5)	O2—Sm1—O5—C16	−95.10 (17)
O6—C12—C13—C14	−178.6 (3)	O7—Sm1—O5—C16	75.38 (17)
C12—C13—C14—C9	−1.0 (5)	O1—Sm1—O5—C16	−73.45 (17)
C10—C9—C14—C13	0.1 (5)	O8—Sm1—O5—C16	122.91 (17)
C15—C9—C14—C13	179.6 (3)	N1—Sm1—O5—C16	0.9 (2)
C10—C9—C15—C16	−99.8 (4)	C8—Sm1—O5—C16	−81.26 (17)
C14—C9—C15—C16	80.6 (4)	C24—Sm1—O5—C16	99.07 (17)
C9—C15—C16—O4	172.7 (3)	O8—C24—O7—Sm1	−7.9 (3)
C9—C15—C16—O5	−4.9 (4)	C23—C24—O7—Sm1	170.0 (3)
C9—C15—C16—Sm1	88.5 (10)	O5—Sm1—O7—C24	71.33 (19)
O5—Sm1—C16—O4	170.2 (3)	O4—Sm1—O7—C24	121.8 (2)
O1W—Sm1—C16—O4	95.9 (3)	O1W—Sm1—O7—C24	−74.19 (19)
O10—Sm1—C16—O4	−178.23 (17)	O10—Sm1—O7—C24	−2.5 (2)
O2—Sm1—C16—O4	−87.59 (18)	O2—Sm1—O7—C24	−121.23 (19)
O7—Sm1—C16—O4	64.78 (18)	O1—Sm1—O7—C24	142.98 (18)
O1—Sm1—C16—O4	−91.43 (18)	O8—Sm1—O7—C24	4.32 (17)
O8—Sm1—C16—O4	115.67 (18)	N1—Sm1—O7—C24	−158.8 (2)
N1—Sm1—C16—O4	−9.11 (19)	C16—Sm1—O7—C24	97.2 (2)
C8—Sm1—C16—O4	−87.17 (18)	C8—Sm1—O7—C24	−164.0 (2)
C24—Sm1—C16—O4	89.63 (18)	O7—C24—O8—Sm2	164.8 (3)
O4—Sm1—C16—O5	−170.2 (3)	C23—C24—O8—Sm2	−13.1 (6)
O1W—Sm1—C16—O5	−74.3 (3)	Sm1—C24—O8—Sm2	157.3 (4)
O10—Sm1—C16—O5	11.61 (17)	O7—C24—O8—Sm1	7.5 (3)
O2—Sm1—C16—O5	102.25 (17)	C23—C24—O8—Sm1	−170.4 (3)
O7—Sm1—C16—O5	−105.38 (17)	O14—Sm2—O8—C24	−82.6 (4)
O1—Sm1—C16—O5	98.40 (17)	O13—Sm2—O8—C24	−74.7 (4)
O8—Sm1—C16—O5	−54.49 (17)	O2W—Sm2—O8—C24	124.6 (4)
N1—Sm1—C16—O5	−179.27 (16)	O16—Sm2—O8—C24	47.4 (4)
C8—Sm1—C16—O5	102.66 (17)	O17—Sm2—O8—C24	−1.6 (4)
C24—Sm1—C16—O5	−80.53 (17)	O10—Sm2—O8—C24	−155.1 (4)
O5—Sm1—C16—C15	−99.6 (10)	O11—Sm2—O8—C24	−170.8 (4)
O4—Sm1—C16—C15	90.2 (10)	N3—Sm2—O8—C24	76.5 (5)
O1W—Sm1—C16—C15	−173.9 (9)	C40—Sm2—O8—C24	−78.0 (4)
O10—Sm1—C16—C15	−88.0 (10)	C48—Sm2—O8—C24	23.9 (4)
O2—Sm1—C16—C15	2.7 (10)	C32—Sm2—O8—C24	−165.7 (4)
O7—Sm1—C16—C15	155.0 (10)	O14—Sm2—O8—Sm1	72.61 (11)
O1—Sm1—C16—C15	−1.2 (10)	O13—Sm2—O8—Sm1	80.48 (8)
O8—Sm1—C16—C15	−154.1 (10)	O2W—Sm2—O8—Sm1	−80.24 (9)
N1—Sm1—C16—C15	81.1 (10)	O16—Sm2—O8—Sm1	−157.40 (9)
C8—Sm1—C16—C15	3.1 (10)	O17—Sm2—O8—Sm1	153.60 (8)
C24—Sm1—C16—C15	179.7 (12)	O10—Sm2—O8—Sm1	0.08 (6)
C22—C17—C18—C19	0.3 (4)	O11—Sm2—O8—Sm1	−15.60 (10)
C23—C17—C18—C19	178.9 (3)	N3—Sm2—O8—Sm1	−128.35 (14)
C17—C18—C19—C20	0.0 (5)	C40—Sm2—O8—Sm1	77.21 (9)
C18—C19—C20—O9	−180.0 (3)	C48—Sm2—O8—Sm1	179.11 (9)
C18—C19—C20—C21	−0.5 (5)	C32—Sm2—O8—Sm1	−10.57 (9)
O9—C20—C21—C22	−179.9 (3)	O5—Sm1—O8—C24	−113.14 (17)
C19—C20—C21—C22	0.6 (5)	O4—Sm1—O8—C24	−66.49 (17)
C18—C17—C22—C21	−0.2 (5)	O1W—Sm1—O8—C24	83.36 (17)
C23—C17—C22—C21	−178.8 (3)	O10—Sm1—O8—C24	169.19 (18)
C20—C21—C22—C17	−0.2 (5)	O2—Sm1—O8—C24	121.15 (17)
C22—C17—C23—C24	98.0 (4)	O7—Sm1—O8—C24	−4.17 (17)
C18—C17—C23—C24	−80.5 (4)	O1—Sm1—O8—C24	−143.66 (18)
C17—C23—C24—O7	−45.2 (5)	N1—Sm1—O8—C24	15.45 (19)
C17—C23—C24—O8	132.7 (3)	C16—Sm1—O8—C24	−90.90 (17)
O5—Sm1—C24—O7	−109.12 (19)	C8—Sm1—O8—C24	162.1 (2)
O4—Sm1—C24—O7	−56.05 (19)	O5—Sm1—O8—Sm2	77.59 (8)
O1W—Sm1—C24—O7	99.17 (19)	O4—Sm1—O8—Sm2	124.24 (8)
O10—Sm1—C24—O7	177.77 (19)	O1W—Sm1—O8—Sm2	−85.92 (9)
O2—Sm1—C24—O7	93.0 (2)	O10—Sm1—O8—Sm2	−0.08 (7)
O1—Sm1—C24—O7	−85.8 (3)	O2—Sm1—O8—Sm2	−48.12 (13)
O8—Sm1—C24—O7	−172.3 (3)	O7—Sm1—O8—Sm2	−173.44 (12)
N1—Sm1—C24—O7	20.6 (2)	O1—Sm1—O8—Sm2	47.06 (16)
C16—Sm1—C24—O7	−82.69 (19)	N1—Sm1—O8—Sm2	−153.82 (8)
O5—Sm1—C24—O8	63.22 (16)	C16—Sm1—O8—Sm2	99.83 (9)
O4—Sm1—C24—O8	116.29 (16)	C8—Sm1—O8—Sm2	−7.1 (2)
O1W—Sm1—C24—O8	−88.49 (17)	C24—Sm1—O8—Sm2	−169.3 (2)
O10—Sm1—C24—O8	−9.89 (17)	O11—C32—O10—Sm1	−151.8 (2)
O2—Sm1—C24—O8	−94.70 (19)	C31—C32—O10—Sm1	28.0 (4)
O7—Sm1—C24—O8	172.3 (3)	Sm2—C32—O10—Sm1	−139.1 (3)
O1—Sm1—C24—O8	86.5 (3)	O11—C32—O10—Sm2	−12.7 (3)
N1—Sm1—C24—O8	−167.03 (16)	C31—C32—O10—Sm2	167.1 (2)
C16—Sm1—C24—O8	89.66 (17)	O5—Sm1—O10—C32	53.5 (3)
C30—C25—C26—C27	−1.0 (4)	O4—Sm1—O10—C32	47.1 (3)
C31—C25—C26—C27	177.9 (3)	O1W—Sm1—O10—C32	−148.6 (3)
C25—C26—C27—C28	−0.5 (5)	O2—Sm1—O10—C32	−73.2 (3)
C26—C27—C28—O12	−179.6 (3)	O7—Sm1—O10—C32	140.8 (3)
C26—C27—C28—C29	1.5 (5)	O1—Sm1—O10—C32	−21.8 (3)
C27—C28—C29—C30	−1.0 (5)	O8—Sm1—O10—C32	135.0 (3)
O12—C28—C29—C30	−179.9 (3)	N1—Sm1—O10—C32	−106.1 (3)
C28—C29—C30—C25	−0.6 (5)	C16—Sm1—O10—C32	48.1 (3)
C26—C25—C30—C29	1.6 (5)	C8—Sm1—O10—C32	−47.8 (3)
C31—C25—C30—C29	−177.3 (3)	C24—Sm1—O10—C32	139.7 (3)
C30—C25—C31—C32	75.7 (4)	O5—Sm1—O10—Sm2	−81.44 (8)
C26—C25—C31—C32	−103.2 (3)	O4—Sm1—O10—Sm2	−87.86 (10)
C25—C31—C32—O11	12.0 (5)	O1W—Sm1—O10—Sm2	76.42 (8)
C25—C31—C32—O10	−167.8 (3)	O2—Sm1—O10—Sm2	151.85 (8)
C25—C31—C32—Sm2	−94.3 (9)	O7—Sm1—O10—Sm2	5.86 (11)
O8—Sm2—C32—O11	−169.19 (17)	O1—Sm1—O10—Sm2	−156.70 (8)
O14—Sm2—C32—O11	62.78 (17)	O8—Sm1—O10—Sm2	0.07 (6)
O13—Sm2—C32—O11	115.67 (17)	N1—Sm1—O10—Sm2	118.99 (15)
O2W—Sm2—C32—O11	−91.42 (18)	C16—Sm1—O10—Sm2	−86.88 (9)
O16—Sm2—C32—O11	−93.80 (19)	C8—Sm1—O10—Sm2	177.23 (9)
O17—Sm2—C32—O11	70.9 (3)	C24—Sm1—O10—Sm2	4.80 (9)
O10—Sm2—C32—O11	167.4 (3)	O8—Sm2—O10—C32	−154.56 (18)
N3—Sm2—C32—O11	−11.50 (18)	O14—Sm2—O10—C32	74.28 (16)
C40—Sm2—C32—O11	89.15 (17)	O13—Sm2—O10—C32	125.87 (17)
O8—Sm2—C32—O10	23.39 (16)	O2W—Sm2—O10—C32	−71.46 (16)
O14—Sm2—C32—O10	−104.64 (16)	O16—Sm2—O10—C32	−116.85 (17)
O13—Sm2—C32—O10	−51.76 (16)	O17—Sm2—O10—C32	140.59 (18)
O2W—Sm2—C32—O10	101.15 (16)	O11—Sm2—O10—C32	6.88 (16)
O16—Sm2—C32—O10	98.78 (18)	N3—Sm2—O10—C32	1.30 (19)
O17—Sm2—C32—O10	−96.5 (3)	C40—Sm2—O10—C32	99.62 (17)
O11—Sm2—C32—O10	−167.4 (3)	C48—Sm2—O10—C32	−157.3 (2)
N3—Sm2—C32—O10	−178.93 (15)	O8—Sm2—O10—Sm1	−0.08 (7)
C40—Sm2—C32—O10	−78.28 (16)	O14—Sm2—O10—Sm1	−131.24 (8)
O8—Sm2—C32—C31	−56.4 (9)	O13—Sm2—O10—Sm1	−79.65 (8)
O14—Sm2—C32—C31	175.6 (9)	O2W—Sm2—O10—Sm1	83.02 (8)
O13—Sm2—C32—C31	−131.5 (9)	O16—Sm2—O10—Sm1	37.63 (13)
O2W—Sm2—C32—C31	21.4 (9)	O17—Sm2—O10—Sm1	−64.93 (16)
O16—Sm2—C32—C31	19.0 (10)	O11—Sm2—O10—Sm1	161.36 (12)
O17—Sm2—C32—C31	−176.3 (8)	N3—Sm2—O10—Sm1	155.78 (8)
O10—Sm2—C32—C31	−79.8 (9)	C40—Sm2—O10—Sm1	−105.90 (8)
O11—Sm2—C32—C31	112.8 (10)	C48—Sm2—O10—Sm1	−2.8 (3)
N3—Sm2—C32—C31	101.3 (9)	C32—Sm2—O10—Sm1	154.5 (2)
C40—Sm2—C32—C31	−158.0 (9)	O10—C32—O11—Sm2	12.5 (3)
C38—C33—C34—C35	−0.1 (5)	C31—C32—O11—Sm2	−167.3 (3)
C39—C33—C34—C35	179.2 (3)	O8—Sm2—O11—C32	11.97 (19)
C33—C34—C35—C36	−0.4 (5)	O14—Sm2—O11—C32	−112.65 (18)
C34—C35—C36—O15	−179.4 (3)	O13—Sm2—O11—C32	−66.94 (18)
C34—C35—C36—C37	0.3 (5)	O2W—Sm2—O11—C32	80.11 (18)
O15—C36—C37—C38	179.9 (3)	O16—Sm2—O11—C32	119.63 (18)
C35—C36—C37—C38	0.2 (5)	O17—Sm2—O11—C32	−149.20 (17)
C34—C33—C38—C37	0.6 (5)	O10—Sm2—O11—C32	−7.00 (16)
C39—C33—C38—C37	−178.7 (3)	N3—Sm2—O11—C32	168.36 (18)
C36—C37—C38—C33	−0.6 (5)	C40—Sm2—O11—C32	−89.78 (18)
C38—C33—C39—C40	−114.4 (3)	C48—Sm2—O11—C32	161.98 (19)
C34—C33—C39—C40	66.3 (4)	O14—C40—O13—Sm2	1.1 (3)
C33—C39—C40—O14	173.8 (3)	C39—C40—O13—Sm2	−177.3 (2)
C33—C39—C40—O13	−7.8 (4)	O8—Sm2—O13—C40	−172.88 (18)
O8—Sm2—C40—O14	−171.84 (16)	O14—Sm2—O13—C40	−0.63 (16)
O13—Sm2—C40—O14	−178.9 (3)	O2W—Sm2—O13—C40	−135.63 (18)
O2W—Sm2—C40—O14	−83.0 (2)	O16—Sm2—O13—C40	115.71 (17)
O16—Sm2—C40—O14	100.50 (17)	O17—Sm2—O13—C40	84.53 (17)
O17—Sm2—C40—O14	92.67 (17)	O10—Sm2—O13—C40	−102.81 (17)
O10—Sm2—C40—O14	−106.64 (17)	O11—Sm2—O13—C40	−59.52 (17)
O11—Sm2—C40—O14	−56.52 (17)	N3—Sm2—O13—C40	21.7 (2)
N3—Sm2—C40—O14	18.84 (18)	C48—Sm2—O13—C40	97.69 (17)
C48—Sm2—C40—O14	94.02 (17)	C32—Sm2—O13—C40	−82.41 (17)
C32—Sm2—C40—O14	−81.54 (17)	O13—C40—O14—Sm2	−1.1 (3)
O8—Sm2—C40—O13	7.03 (17)	C39—C40—O14—Sm2	177.3 (2)
O14—Sm2—C40—O13	178.9 (3)	O8—Sm2—O14—C40	10.1 (2)
O2W—Sm2—C40—O13	95.9 (2)	O13—Sm2—O14—C40	0.64 (16)
O16—Sm2—C40—O13	−80.64 (18)	O2W—Sm2—O14—C40	140.64 (16)
O17—Sm2—C40—O13	−88.47 (17)	O16—Sm2—O14—C40	−99.49 (17)
O10—Sm2—C40—O13	72.22 (16)	O17—Sm2—O14—C40	−80.88 (17)
O11—Sm2—C40—O13	122.35 (17)	O10—Sm2—O14—C40	71.96 (17)
N3—Sm2—C40—O13	−162.29 (16)	O11—Sm2—O14—C40	119.62 (17)
C48—Sm2—C40—O13	−87.11 (17)	N3—Sm2—O14—C40	−160.90 (18)
C32—Sm2—C40—O13	97.32 (17)	C48—Sm2—O14—C40	−91.52 (17)
C46—C41—C42—C43	0.0 (4)	C32—Sm2—O14—C40	96.54 (17)
C47—C41—C42—C43	177.1 (3)	O17—C48—O16—Sm2	9.3 (3)
C41—C42—C43—C44	−1.3 (5)	C47—C48—O16—Sm2	−169.6 (2)
C42—C43—C44—O18	−178.4 (3)	O8—Sm2—O16—C48	−112.93 (16)
C42—C43—C44—C45	1.9 (5)	O14—Sm2—O16—C48	18.30 (19)
O18—C44—C45—C46	179.2 (3)	O13—Sm2—O16—C48	−44.99 (17)
C43—C44—C45—C46	−1.3 (5)	O2W—Sm2—O16—C48	166.54 (17)
C44—C45—C46—C41	−0.1 (5)	O17—Sm2—O16—C48	−5.15 (15)
C42—C41—C46—C45	0.7 (4)	O10—Sm2—O16—C48	−147.80 (15)
C47—C41—C46—C45	−176.5 (3)	O11—Sm2—O16—C48	127.73 (16)
C42—C41—C47—C48	−69.6 (4)	N3—Sm2—O16—C48	79.21 (16)
C46—C41—C47—C48	107.4 (3)	C40—Sm2—O16—C48	−14.81 (19)
C41—C47—C48—O16	57.3 (4)	C32—Sm2—O16—C48	168.84 (15)
C41—C47—C48—O17	−121.6 (3)	O16—C48—O17—Sm2	−9.0 (3)
O8—Sm2—C48—O16	65.60 (16)	C47—C48—O17—Sm2	169.9 (2)
O14—Sm2—C48—O16	−165.05 (15)	O8—Sm2—O17—C48	77.00 (17)
O13—Sm2—C48—O16	140.81 (15)	O14—Sm2—O17—C48	−155.86 (17)
O2W—Sm2—C48—O16	−13.28 (17)	O13—Sm2—O17—C48	149.37 (18)
O17—Sm2—C48—O16	170.8 (3)	O2W—Sm2—O17—C48	−5.1 (2)
O10—Sm2—C48—O16	68.1 (3)	O16—Sm2—O17—C48	5.04 (15)
O11—Sm2—C48—O16	−87.5 (2)	O10—Sm2—O17—C48	134.67 (16)
N3—Sm2—C48—O16	−93.97 (16)	O11—Sm2—O17—C48	−120.14 (17)
C40—Sm2—C48—O16	167.87 (16)	N3—Sm2—O17—C48	−78.00 (17)
O8—Sm2—C48—O17	−105.24 (17)	C40—Sm2—O17—C48	176.99 (18)
O14—Sm2—C48—O17	24.12 (17)	C32—Sm2—O17—C48	−164.1 (2)

### Hydrogen-bond geometry (Å, °)

**Table:**

*D*—H···*A*	*D*—H	H···*A*	*D*···*A*	*D*—H···*A*
O3—H3B···O12^i^	0.82	1.94	2.749 (3)	169
O6—H6B···O3W^ii^	0.82	1.86	2.642 (3)	159
O9—H9A···O17^iii^	0.82	1.86	2.673 (3)	173
O12—H12A···O11^iv^	0.82	1.94	2.746 (3)	168
O15—H15C···O6^v^	0.82	1.91	2.722 (3)	174
O18—H18B···O9^ii^	0.82	1.95	2.768 (3)	173
O2W—H2WA···O5	0.83 (5)	2.02 (5)	2.767 (3)	150 (4)
O2W—H2WB···N2^ii^	0.83 (2)	2.03 (2)	2.841 (3)	164 (5)
O3W—H3WB···O3	0.85 (4)	1.99 (2)	2.816 (3)	167 (5)
O1W—H1WA···O13	0.81 (5)	1.96 (4)	2.755 (3)	165 (4)
O1W—H1WB···N4^i^	0.83 (2)	1.99 (2)	2.778 (3)	160 (4)
O3W—H3WA···O1^vi^	0.84 (4)	1.94 (4)	2.773 (3)	172 (4)

Symmetry codes: (i) *x*, *y*+1, *z*; (ii) *x*, *y*−1, *z*; (iii) −*x*, −*y*+1, −*z*; (iv) −*x*, −*y*, −*z*+1; (v) *x*−1, *y*+1, *z*; (vi) −*x*+1, −*y*+1, −*z*+1.
